# High-throughput sequencing of virus-infected *Cucurbita pepo* samples revealed the presence of Zucchini shoestring virus in Zimbabwe

**DOI:** 10.1186/s13104-020-4927-3

**Published:** 2020-02-03

**Authors:** Charles Karavina, Jacques Davy Ibaba, Augustine Gubba

**Affiliations:** 1 Department of Crop Science, Bindura University of Science Education, Astra Campus, P. Bag 1020, Bindura, Zimbabwe; 2 Discipline of Plant Pathology, University of KwaZulu-Natal, Agriculture Campus, 1 Agric Avenue, Scottsville, Pietermaritzburg, 3209 Republic of South Africa

**Keywords:** Next generation sequencing, Potyvirus, Plant virus, Cucurbit, Zimbabwe, Zucchini shoestring virus, High-throughput sequencing

## Abstract

**Objectives:**

Plant-infecting viruses remain a serious challenge towards achieving food security worldwide. Cucurbit virus surveys were conducted in Zimbabwe during the 2014 and 2015 growing seasons. Leaf samples displaying virus-like symptoms were collected and stored until analysis. Three baby marrow samples were subjected to next-generation sequencing and the data generated were analysed using genomics technologies. Zucchini shoestring virus (ZSSV), a cucurbit-infecting potyvirus previously described in South Africa was one of the viruses identified. The genomes of the three ZSSV isolates are described analysed in this note.

**Results:**

The three ZSSV isolates had the same genome size of 10,297 bp excluding the polyA tail with a 43% GC content. The large open reading frame was found at positions 69 to 10,106 on the genome and encodes a 3345 amino acids long polyprotein which had the same cleavage site sequences as those described on the South African isolate except for the P1-pro site. Genome sequence comparisons of all the ZSSV isolates showed that the isolates F7-Art and S6-Prime had identical sequence across the entire genome while sharing 99.06% and 99.34% polyprotein nucleotide and amino acid sequence identities, respectively with the isolate S7-Prime.

## Introduction

Cucurbit is a generic term used to denote all species within the Family *Cucurbitaceae* also know as the gourd family [1]. Numerous cucurbit crops are economically important worldwide. Cucurbits are consumed in different ways as fruits or vegetables, providing essential nutrients and dietary fibre [2]. In Zimbabwe, Some of the cultivated cucurbits include the cucumber (*Cucumis melo* L.), the watermelon (*Citrullus lanatus* (Thunb.) Matsum. & Nakai), the melon (*Cucumis melo* L.), the pumpkin (*Cucurbita maxima* Duch.), the butternut (*Cucurbita moschata* Duch.) and the baby marrow (*Cucurbita pepo* L.). They are widely grown by both commercial and smallholder farmers as food and cash crops. Virus diseases on cucurbits produce diverse symptoms that result in yield reduction and in severe instances compromised fruit quality [3, 4]. The negative effects of plant-infecting viruses on crops are more prominent especially in countries where their studies are underdeveloped.

High-throughput sequencing (HTS), also called next-generation sequencing (NGS) describes a series of technologies whereby millions or billions of DNA molecules are sequenced simultaneously [5]. The application of these ever-growing sequencing technologies and bioinformatics data analysis to the studies of plant-infecting viruses, which started in 2009 [5], have revolutionized the fields of virus discovery and diagnostics, resulting in unprecedented virus discoveries from any host and environment [6]. Unlike other popular techniques such as the enzyme-linked immunosorbent assay, molecular hybridization and polymerase chain reaction that mainly work on known pathogens, HTS data analysis has made possible the identification of sequences of known or unknown viruses from any host without any prior knowledge of the disease aetiology [7, 8].

Zucchini shoestring virus (ZSSV) was discovered among other known cucurbit-infecting viruses in 2015 in South Africa when the RNA from severely distorted Baby marrow leaves were subjected to HTS [9, 10]. Genomics and taxonomic studies revealed that ZSSV is a new species in the genus *Potyvirus* [10]. The International Committee TV subsequently ratified these findings [11]. The genus *Potyvirus* is one of the 8 genera that composed the family *Potyviridae*. Members in that family, also known as potyvirids, are differentiated by the host range, genomic features and phylogeny, with a species demarcation criterion set to a nucleotide and amino sequence identity less than 76% and 82%, respectively for the large open reading frame (ORF) or its protein product. In instances where the complete ORF sequence is not available, similar criteria can be used for the coat protein (CP) coding region [12].

Viruses that belong to the genus *Potyvirus* have non-enveloped, flexuous and filamentous virions of 680–900 nm in length and 11–20 nm in diameter. The genome of potyviruses is a positive-sense ssRNA molecule with its 5′ terminus covalently linked to the viral protein genome linked (VPg) and its 3′ end polyadenylated. The 10,000 bp genome harbours two ORFs that encode eleven multifunctional proteins. A large ORF is translated into a single polyprotein that is cleaved at semi-conserved sites by three self-encoded proteases into ten mature proteins namely the protein 1 protease (P1-Pro), the helper component proteinase (HC-Pro), Protein 3 (P3), six kilodalton peptide 1 (6K1), the 6K2, the cytoplasmic inclusion (CI), the nuclear inclusion A protease (NIa-Pro), the nuclear inclusion B RNA-dependent RNA polymerase (NIb), the VPg and the CP [12]. A smaller ORF, named the pretty interesting *Potyviridae* ORF (PIPO), is generated by a polymerase slippage mechanism and is expressed as the trans-frame protein P3N-PIPO [13–15].

In this note, we described and studied the genome sequences of three ZSSV isolates obtained through HTS of infected baby marrow leaves collected in Zimbabwe.

## Main text

### Sample sources

**Fig. 1:**
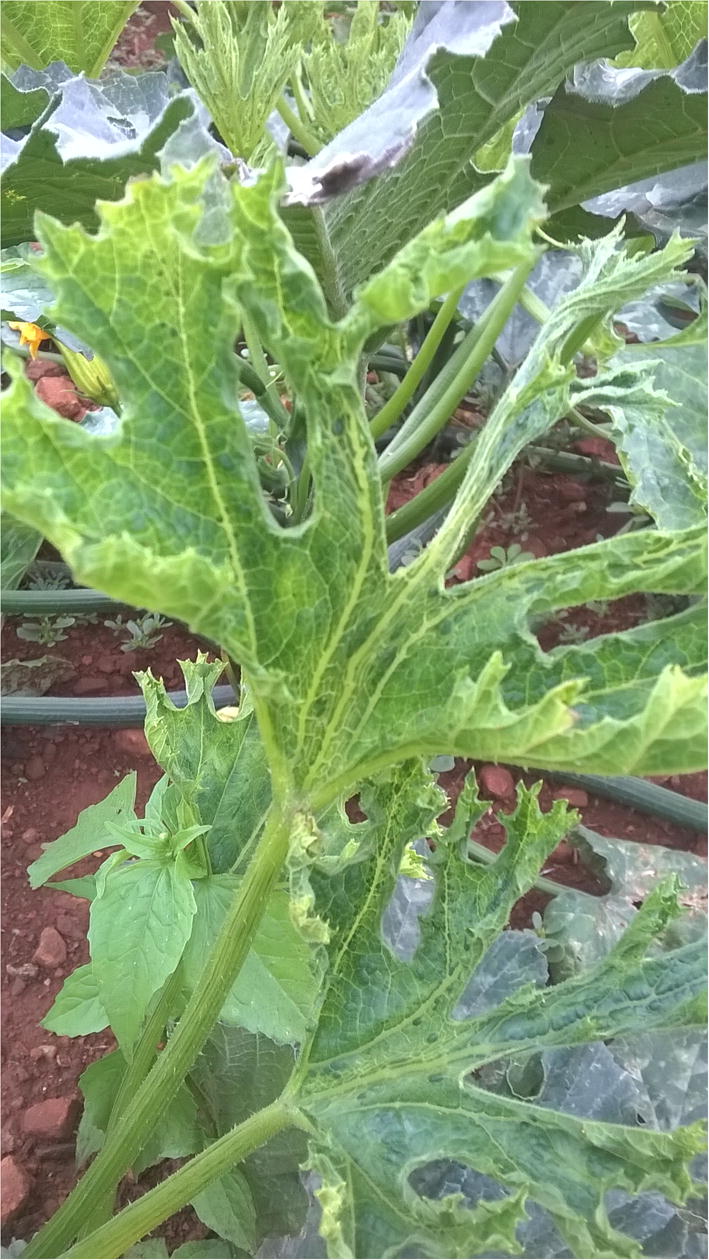
Picture of the most common symptom observed on baby marrow plants during the survey conducted in selected cucurbit-growing farms in Harare in 2014 and 2015

Virus surveys were conducted in selected cucurbit farms in Harare, Zimbabwe, in 2014 and 2015 growing seasons. Baby marrow plants (C*ucurbita pepo*) displaying mosaic and mild leaf distortion (Fig. 1) were the most prevalent symptoms of viral aetiology observed throughout the surveys. Labelled samples were collected and consisted of one symptomatic younger leaf fully developed preserved in RNAlater Solution (ThermoFisher Scientific, USA). Three leaf samples from three different farms were randomly selected for HTS.

### High-throughput sequencing and data analysis

Total RNA was extracted from each leaf sample using the Quick-RNA Miniprep Kit (Zymo Research, USA) as per the manufacturer’s instructions and was shipped on dry ice to the Agricultural Research Council Biotechnology Platform (ARC-BTP) in Pretoria, South Africa for sequencing on the HiSeq platform (Illumina Inc., USA). For each sample, the data generated from sequencing was analysed as follows. The read quality was assessed using FastQC version 0.11.5 (Babraham Bioinformatics) and when necessary, Trimmomatic version 0.36 [16] was used to trim. De novo assembly was then performed using SPAdes version 3.10.1 [17] according to the developer’s instructions. Nucleotide blast was performed on all contig using BLAST+ [18].

### Genomics and phylogenetic analysis

The ORFfinder web version (https://www.ncbi.nlm.nih.gov/orffinder/) was used to identify ORFs. ClustalW [19] was used to do multiple sequence alignment. Nucleotide and amino acid sequence identities were performed online with SIAS (http://imed.med.ucm.es/Tools/sias.html). MEGA X software version 10.1.7 [20] was used to find the best evolutionary model fitting our phylogenetic analysis and to infer the maximum likelihood tree accordingly. ZSSV being one of the species in the “Papaya ringspot virus (PRSV) cluster” of cucurbit-infecting potyviruses, the phylogenetic analyses were performed using the CP coding sequences of selected members of this cluster.

### Results

#### ZSSV genome sequence identified from HTS data analysis

The BLAST results identified one contig from each sample as a perfect match to the full-length genome sequence of the South African (SA) ZSSV isolate (GenBank accession number: KU355553.1). These sequences were then referred as ZSSS isolates F7-Art, S6-Prime and S7-Prime. The coverage values were 30×, 66× and 80× for F7-Art, S6-Prime and S7-Prime, respectively. The genome size was the same for the three isolates and consisted of 10,297 bp excluding the polyA tail with GC contents varying between 42.92 and 42.96%. Each isolate sequence was submitted to GenBank and was given accession number as surmised in Table 1.

**Table 1:** GenBank accession number of the ZSSV isolates described in this study

Isolate name	Genbank accession number
Zucchini shoestring virus isolate F7-Art	MK204479.1
Zucchini shoestring virus isolate S6-Prime	MK204480.1
Zucchini shoestring virus isolate S7-Prime	MK204481.1

#### ZSSV genome analysis and phylogeny

The genome features common to the three isolates included the lengths and the positions of both ORFs and the polyprotein cleavage site sequences. The large ORF was located at positions 69 to 10,106 of the genome. The polyprotein resulting from the direct translation of the large ORF was 3345 amino acids long. The PIPO ORF was situated from nucleotide position 3611 to 3793. The LAIGN box that has been reported to play a role in virus movement and amplification [21] and the FRNK box involved in RNA silencing and symptom development [22] were identified on the HC-Pro of all the ZSSV isolates. The motifs DAG [23], RITC and PTR involved in aphid transmission were also part of the CP and the HC-Pro.

The polyprotein cleavage site sequences of the three isolates described in this study were the same as the SA isolate [10] except for the P1-pro site that was IVHY|S instead of IIHY|S. Genome sequence comparisons of all the ZSSV isolates are available in Additional files 1 and 2. They showed that the isolates F7-Art and S6-Prime had identical sequence across the entire genome while sharing 99.06% and 99.34% polyprotein nucleotide and amino acid sequence identities, respectively with the isolate S7-Prime. The CP, 6K1, 6K2 and 5′ terminus nucleotide and amino acid sequences were the same for the three isolates under study. The amino acid sequence of the HC-Pro and the NIa-Pro were 100% identical although their corresponding nucleotide sequences were not. The lowest percentage values of 97.78% and 97.21% were recorded with the P1-Pro nucleotide and amino acid sequence, respectively. When compared with the SA isolate, the polyprotein nucleotide sequence identities was 91.08% with the isolates F7-Art and 92.02 with the isolate S7-Prime. The polyprotein amino acid sequence identities percentages were a bit higher at 95.84% and 96.5% against the isolates F7-Art and the isolate S7-Prime, respectively. At the individual genome features nucleotide and amino acid sequence identity between the SA isolate and the ZSSV isolates from Zimbabwe ranged from 87.87 to 96.39% and from 87.1 to 99.34%, respectively.

**Fig. 2:**
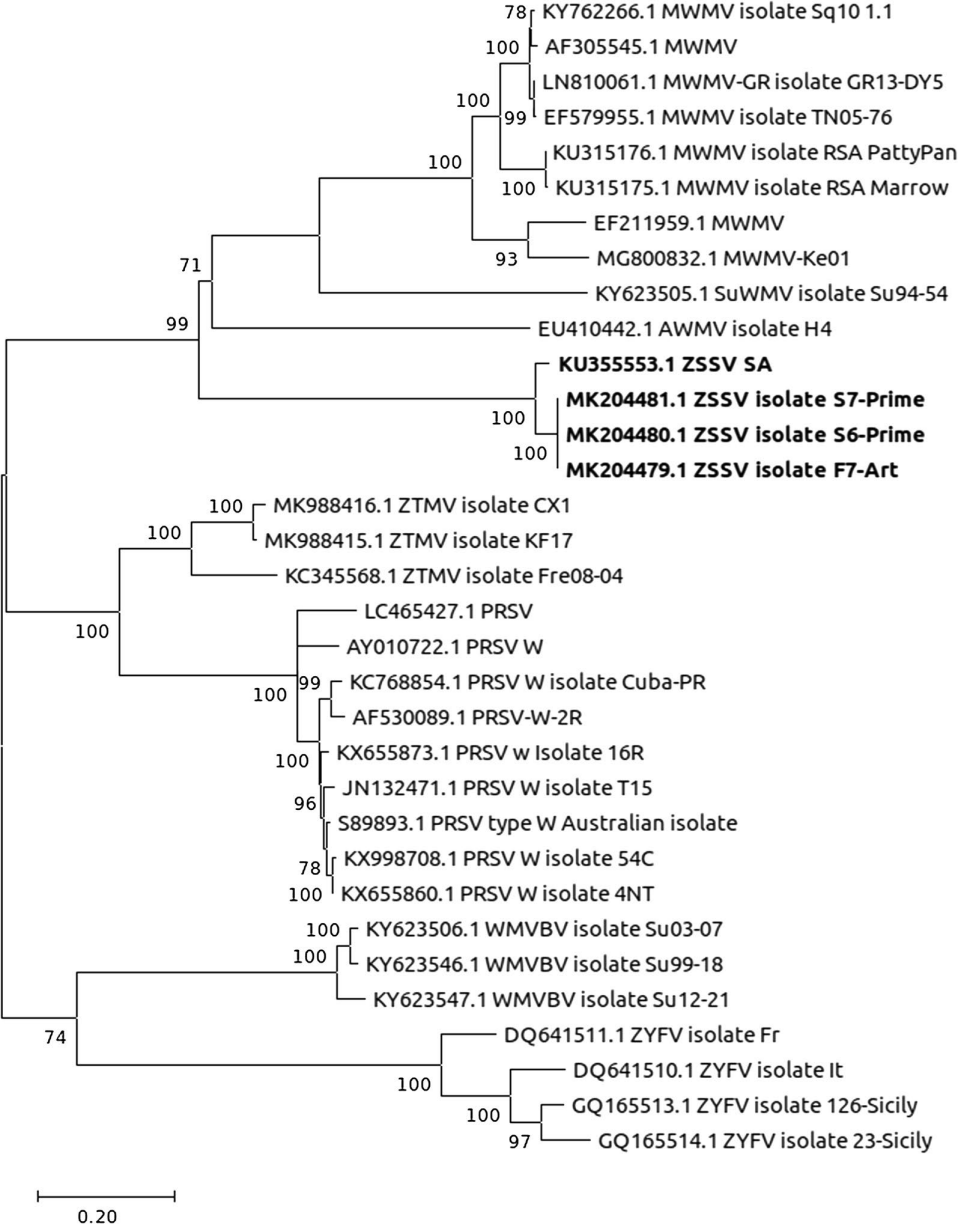
Maximum likelihood tree of selected members of the PRSV cluster of cucurbit-infecting viruses. The bootstrap percentage values are shown next to the branches. The tree is drawn to scale, with branch lengths measured in the number of substitutions per site. MWMV: Moroccan watermelon mosaic virus; SuWMV: Sudan watermelon mosaic virus; AWMV: Algerian watermelon mosaic virus; ZSSV: Zucchini shoestring virus; ZTMV: Zucchini tigré mosaic virus; PRSV: Papaya ringspot virus; WMVBV: Wild melon vein banding virus; ZYFV: Zucchini yellow fleck virus

The phylogenetic analysis involved 33 nucleotide sequences and was inferred using the general time-reversible model with a discrete Gamma distribution (5 categories (+G, parameter = 0.8565)) and invariable sites ([+I], 27.21% sites). The tree with superior log-likelihood value (− 9554.87) was automatically selected (Fig. 2). The selected isolates in the tree were divided into three main groups. One group was made of Moroccan watermelon mosaic virus (MWMV) isolates, Sudan watermelon mosaic virus (SuWMV) isolates, Algerian watermelon mosaic virus (AWMV) isolates and ZSSV isolates. In another group were included Zucchini tigré mosaic virus (ZTMV) isolates and PRSV isolates. The last group comprised Wild melon vein banding virus (WMVBV) isolates and Zucchini yellow fleck virus (ZYFV) isolates. All the ZSSV isolates clustered together with 100% bootstrap value.

### Discussion

PRSV cluster of curcurbit-infecting virus include eight acknowledged species. Four of those species, ZTMV [24], ZSSV [10], SuWMV and WMVBV [25], have been reported in the past 7 years. Moreover, MWMV, AWMV, ZSSV, SuWMV and WMVBV were identified in Africa, suggesting that the PRSV cluster underwent an important diversification in Africa [25]. Out of these viruses present in Africa, MWMV is the widespread one having been reported in all African regions [3, 26–32]. The HTS in this study made the detection of ZSSV on infected leaf sample possible. The presence of ZSSV in cultivated baby marrow plants from the surveyed farms may indicate either a broader geographical distribution of the virus or its spreading across borders. The occurrence of ZZSV in Zimbabwe highlights the need to conduct further studies on its epidemiology and to develop effective management strategies.

## Limitations

1. The small number of samples analysed in that study was one of the limitations.
2. ZSSV at this stage of the study can not be considered the main causal agent of the symptoms identified in the virus surveys.

## Supplementary information

**Additional file 1.** Nucleotide sequence identities of the Zucchini shoestring virus (ZSSV) isolates. Table displaying the nucleotide sequence identities in percentage between all ZSSV isolates available on GenBank.

**Additional file 2.** Amino acid sequence identities of the Zucchini shoestring virus (ZSSV) isolates. Table displaying the amino acid sequence identities in percentage between all ZSSV isolates available on GenBank.

## Data Availability

The ZSSV genome sequences generated in this study can be freely and openly accessed on the NCBI GenBank under the Accession Numbers MK204479.1, MK204480.1 and MK204481.1. Please see Table 1 for details and links.

## Abbreviations

AWMV: Algerian watermelon mosaic virus
ARC-BTP: Agricultural Research Council Biotechnology Platform
CI: Cytoplasmic inclusion
CP: Coat protein
HC-Pro: Helper component proteinase
HTS: High-throughput sequencing
MWMV: Moroccan watermelon mosaic virus
NGS: Next generation sequencing
NIa-Pro: Nuclear inclusion A protease
NIb: Nuclear inclusion B RNA-dependent RNA polymerase
ORF: Open reading frame
P1-Pro: Protein 1 protease
P3: Protein 3
PIPO: Pretty interesting *Potyviridae* open reading frame
PRSV: Papaya ringspot virus
RNA: Ribonucleic acid
SA: South Africa
SuWMV: Sudan watermelon mosaic virus
VPg: Viral protein genome-linked
WMVBV: Wild melon vein banding virus
ZTMV: Zucchini tigré mosaic virus
ZSSV: Zucchini shoestring virus
ZYFV: Zucchini yellow fleck virus
6k1: Six kilodalton peptide 1
6k2: Six kilodalton peptide 2
