# Supplementary material for: High-throughput sequencing of virus-infected Cucurbita pepo samples revealed the presence of Zucchini shoestring virus in Zimbabwe
Source: BMC Res Notes. 2020 Feb 3;13:53. doi: 10.1186/s13104-020-4927-3 (PMC6998830; doi:10.1186/s13104-020-4927-3)
Supplement: Supplementary file 1 — Additional file 1. Nucleotide sequence identities of the Zucchini shoestring virus (ZSSV) isolates. Table displaying the nucleotide sequence identities in percentage between all ZSSV isolates available on GenBank. [file 13104_2020_4927_MOESM1_ESM.pdf]

Additional file 1: Nucleotide sequence identities of the Zucchini shoestring virus (ZSSV) isolates

Table displaying the nucleotide sequence identities in percentage between all ZSSV isolates available on GenBank

Genome features	ZSSV isolates	ZSSV isolate SA	ZSSV isolate S7-Prime	ZSSV isolate S6-Prime	ZSSV isolate F7-Art
Polyprotein	ZSSV isolate SA	100%			
	ZSSV isolate S7-Prime	92.02%	100%		
	ZSSV isolate S6-Prime	91.08%	99.06%	100%	
	ZSSV isolate F7-Art	91.08%	99.06%	100%	100%
CP	ZSSV isolate SA	100%			
	ZSSV isolate S7-Prime	95.17%	100%		
	ZSSV isolate S6-Prime	95.17%	100%	100%	
	ZSSV isolate F7-Art	95.17%	100%	100%	100%
CI	ZSSV isolate SA	100%			
	ZSSV isolate S7-Prime	92.11%	100%		
	ZSSV isolate S6-Prime	91.79%	99.68%	100%	
	ZSSV isolate F7-Art	91.79%	99.68%	100%	100%
HC-Pro	ZSSV isolate SA	100%			
	ZSSV isolate S7-Prime	92.19%	100%		
	ZSSV isolate S6-Prime	92.04%	99.85%	100%	
	ZSSV isolate F7-Art	92.04%	99.85%	100%	100%
NIa-Pro	ZSSV isolate SA	100%			
	ZSSV isolate S7-Prime	94.14%	100%		
	ZSSV isolate S6-Prime	93.16%	99.02%	100%	
	ZSSV isolate F7-Art	93.16%	99.02%	100%	100%
NIb	ZSSV isolate SA	100%			
	ZSSV isolate S7-Prime	90.39%	100%		
	ZSSV isolate S6-Prime	88.2%	97.8%	100%	
	ZSSV isolate F7-Art	88.2%	97.8%	100%	100%

P1-Pro	ZSSV isolate SA	100%			
	ZSSV isolate S7-Prime	90.9%	100%		
	ZSSV isolate S6-Prime	88.69%	97.78%	100%	
	ZSSV isolate F7-Art	88.69%	97.78%	100%	100%
P3	ZSSV isolate SA	100%			
	ZSSV isolate S7-Prime	92.15%	100%		
	ZSSV isolate S6-Prime	91.76%	99.61%	100%	
	ZSSV isolate F7-Art	91.76%	99.61%	100%	100%
VPg	ZSSV isolate SA	100%			
	ZSSV isolate S7-Prime	92.8%	100%		
	ZSSV isolate S6-Prime	92.28%	99.47%	100%	
	ZSSV isolate F7-Art	92.28%	99.47%	100%	100%
6K1	ZSSV isolate SA	100%			
	ZSSV isolate S7-Prime	89.1%	100%		
	ZSSV isolate S6-Prime	89.1%	100%	100%	
	ZSSV isolate F7-Art	89.1%	100%	100%	100%
6K2	ZSSV isolate SA	100%			
	ZSSV isolate S7-Prime	92.39%	100%		
	ZSSV isolate S6-Prime	92.39%	100%	100%	
	ZSSV isolate F7-Art	92.39%	100%	100%	100%
5' terminus	ZSSV isolate SA	100%			
	ZSSV isolate S7-Prime	87.87%	100%		
	ZSSV isolate S6-Prime	87.87%	100%	100%	
	ZSSV isolate F7-Art	87.87%	100%	100%	100%
3' terminus	ZSSV isolate SA	100%			
	ZSSV isolate S7-Prime	96.39%	100%		
	ZSSV isolate S6-Prime	95.36%	98.96%	100%	
	ZSSV isolate F7-Art	95.36%	98.96%	100%	100%
