# Supplementary material for: High-throughput sequencing of virus-infected Cucurbita pepo samples revealed the presence of Zucchini shoestring virus in Zimbabwe
Source: BMC Res Notes. 2020 Feb 3;13:53. doi: 10.1186/s13104-020-4927-3 (PMC6998830; doi:10.1186/s13104-020-4927-3)
Supplement: Supplementary file 2 — Additional file 2. Amino acid sequence identities of the Zucchini shoestring virus (ZSSV) isolates. Table displaying the amino acid sequence identities in percentage between all ZSSV isolates available on GenBank. [file 13104_2020_4927_MOESM2_ESM.pdf]

Additional file 2: Amino acid sequence identities of the Zucchini shoestring virus (ZSSV) isolates

Table displaying the amino acid sequence identities in percentage between all ZSSV isolates available on GenBank

Genome features	Isolates	ZSSV isolate RSA	ZSSV isolate F7-Art	ZSSV isolate S6-Prime	ZSSV isolate S7-Prime
Polyprotein	ZSSV isolate SA	100%			
	ZSSV isolate F7-Art	95.84%	100%		
	ZSSV isolate S6-Prime	95.84%	100%	100%	
	ZSSV isolate S7-Prime	96.5%	99.34%	99.34%	100%
CP	ZSSV isolate SA	100%			
	ZSSV isolate F7-Art	97.16%	100%		
	ZSSV isolate S6-Prime	97.16%	100%	100%	
	ZSSV isolate S7-Prime	97.16%	100%	100%	100%
CI	ZSSV isolate SA	100%			
	ZSSV isolate F7-Art	98.73%	100%		
	ZSSV isolate S6-Prime	98.73%	100%	100%	
	ZSSV isolate S7-Prime	98.89%	99.84%	99.84%	100%
HC-Pro	ZSSV isolate SA	100%			
	ZSSV isolate F7-Art	99.34%	100%		
	ZSSV isolate S6-Prime	99.34%	100%	100%	
	ZSSV isolate S7-Prime	99.34%	100%	100%	100%
NIa-Pro	ZSSV isolate SA	100%			
	ZSSV isolate F7-Art	97.48%	100%		
	ZSSV isolate S6-Prime	97.48%	100%	100%	
	ZSSV isolate S7-Prime	97.48%	100%	100%	100%

NIb	ZSSV isolate SA	100%			
	ZSSV isolate F7-Art	97.29%	100%		
	ZSSV isolate S6-Prime	97.29%	100%	100%	
	ZSSV isolate S7-Prime	97.87%	99.41%	99.41%	100%
P1-Pro	ZSSV isolate SA	100%			
	ZSSV isolate F7-Art	87.1%	100%		
	ZSSV isolate S6-Prime	87.1%	100%	100%	
	ZSSV isolate S7-Prime	89.89%	97.21%	97.21%	100%
P3	ZSSV isolate SA	100%			
	ZSSV isolate F7-Art	95.05%	100%		
	ZSSV isolate S6-Prime	95.05%	100%	100%	
	ZSSV isolate S7-Prime	95.34%	99.7%	99.7%	100%
VPg	ZSSV isolate SA	100%			
	ZSSV isolate F7-Art	97.89%	100%		
	ZSSV isolate S6-Prime	97.89%	100%	100%	
	ZSSV isolate S7-Prime	98.42%	99.47%	99.47%	100%
6K1	ZSSV isolate SA	100%			
	ZSSV isolate F7-Art	94.23%	100%		
	ZSSV isolate S6-Prime	94.23%	100%	100%	
	ZSSV isolate S7-Prime	94.23%	100%	100%	100%
6K2	ZSSV isolate SA	100%			
	ZSSV isolate F7-Art	98.24%	100%		
	ZSSV isolate S6-Prime	98.24%	100%	100%	
	ZSSV isolate S7-Prime	98.24%	100%	100%	100%
